# Evaluation of pharmacist-led medication reconciliation at county hospitals in China: A multicentre, open-label, assessor-blinded, nonrandomised controlled study

**DOI:** 10.7189/jogh.14.04058

**Published:** 2024-04-12

**Authors:** Mengyuan Fu, Yuezhen Zhu, Guilin Wei, Aichen Yu, Fanghui Chen, Yuanpeng Tang, Zining Wang, Guoying Wang, Qingpeng Liu, Chunyuan Zhong, Jinghong Liu, Jie Zhong, Ping Tian, Debao Li, Xixi Li, Luwen Shi, Xiaodong Guan

**Affiliations:** 1 Department of Pharmacy Administration and Clinical Pharmacy, School of Pharmaceutical Sciences, Peking University, Beijing, China; 2 International Research Center for Medicinal Administration, Peking University, Beijing, China; 3 Department of Pharmacy, Beijing Chao-Yang Hospital, Capital Medical University, Chaoyang District, Beijing, China; 4 Department of Pharmacy, The First Affiliated Hospital of Gannan Medical University, Jiangxi, China; 5 Department of Pharmacy, Peking University First Hospital, Beijing, China; 6 Department of Pharmacy, The Peoples’ Hospital of Yudu County, Jiangxi, China; 7 Department of Pharmacy, The Peoples’ Hospital of Xingguo County, Jiangxi, China; 8 Department of Pharmacy, The First People’s Hospital of Longnan City, Jiangxi, China; 9 Department of Pharmacy, The People’s Hospital of Ruijin City, Jiangxi, China; 10 Department of Pharmacy, The People’s Hospital of Shangyou County, Jiangxi, China; 11 Department of Pharmacy, The People’s Hospital of Xinfeng County, Jiangxi, China

## Abstract

**Background:**

Due to a lack of related research, we aimed to determine the effectiveness of a pharmacist-led medication reconciliation intervention in China.

**Methods:**

We conducted a multicentre, prospective, open-label, assessor-blinded, cluster, nonrandomised controlled study at six county-level hospitals, with hospital wards serving as the clusters. We included patients discharged from the sampled hospitals who were aged ≥60 years; had ≥1 studied diagnoses; and were prescribed with ≥3 medications at discharge. Patients in the intervention group received a pharmacist-led medication reconciliation intervention and those in the control group received standard care. We assessed the incidence of medication discrepancies at discharge, patients’ medication adherence, and health care utilisation within 30 days after discharge.

**Results:**

There were 429 patients in the intervention group (mean age = 72.5 years, standard deviation (SD) = 7.0) and 526 patients in the control group (mean age = 73.6 years, SD = 7.1). Of the 1632 medication discrepancies identified at discharge, fewer occurred in the intervention group (1.9 per patient on average) than the control group (2.6 per patient on average).The intervention significantly reduced the incidence of medication discrepancy by 9.6% (95% confidence interval (CI) = −15.6, −3.6, *P* = 0.002) and improved patients’ medication adherence, with an absolute decrease in the mean adherence score of 2.5 (95% CI = −2.8, −2.2, *P* < 0.001). There was no significant difference in readmission rates between the intervention and control groups.

**Conclusions:**

Pharmacist-led medication reconciliation at discharge from Chinese county-level hospitals reduced medication discrepancies and improved patients’ adherence among patients aged 60 years or above, though no impact on readmission after discharge was observed.

**Registration:**

ChiCTR2100045668.

Medication discrepancies, generally defined as inconsistencies between two or more medication lists [1,2], are prevalent at care transitions, as patients’ medication information may not be accurately communicated to patients and/or across health facilities [3–5]. According to a previous systematic review, the incidence of medication discrepancies after hospital discharge can range from 14% to 94% [6]. These discrepancies may cause adverse drug events; exacerbate the patient’s clinical conditions; increase hospital readmission rates; and increase the utilisation of medical resources [7]. Elderly patients are at greater risks of such discrepancies and adverse outcomes due to their age-related physiological changes and polypharmacy resulting from multiple comorbidities [4,8].

Several international patient safety organisations, such as the Joint Commission, the Institute for Healthcare Improvement, and the World Health Organization (WHO), acknowledged medication reconciliation as an important process for improving patient safety by minimising the risk of medication discrepancies, especially at care transitions [9]. The WHO High 5s project, a joint commission whose goal is to improve patient safety through the implementation of standardised operating protocols, stated that the implementation of medication reconciliation is most successful when a pharmacist (or pharmacy staff) is available [10]. In fact, studies across developed countries found that pharmacist-led medication reconciliation could reduce medication discrepancies [11]. However, this process is still being piloted in China [12] and only a few single-centre studies evaluated the effect of medication reconciliation at tertiary-level hospitals [13–16]. Meanwhile, county-level hospitals, which are a key component of the three-tier health care delivery structure in rural China, serve more than 70% of residents across the country [17]; they were previously found to have an 83.0% prevalence of medication discrepancies, signalling a clear target for improving patient outcomes [18]. Due to the severity of this issue, we conducted and evaluate the effect of a pharmacists-led medication reconciliation intervention for elderly patients at county-level hospitals in China.

## METHODS

### Study design and setting

We conducted a multicentre, prospective, open-label, assessor-blinded, cluster, nonrandomised controlled study comparing an intervention with standard care within seven county-level hospitals in the city of Ganzhou, province of Jiangxi, from 15 November 2021 to 15 March 2022. Ganzhou is the largest city in the province of Jiangxi; it is located in central China and has a residential population of 9.8 million. We selected two internal medicine wards for cardiovascular and respiratory medicine (which admitted the most elderly patients) from each sampled hospital. We then randomly allocated them to either the intervention or control group in each hospital. Patients in the intervention group received pharmacist-led medication reconciliation at discharge and those in the control group received standard care.

We obtained the ethical approval from the Peking University Institution Review Board (IRB00001052-21016) and all participating county-level hospitals. We performed the study and reported our findings per the CONSORT guidelines [19,20], registered it with the Chinese Clinical Trial Registry (ChiCTR2100045668), and published the protocol elsewhere [21].

### Participants

Patients who were treated and discharged from the sampled wards were eligible if they were aged ≥60 years; had at least one of the following diagnoses: hypertension, hyperlipidaemia, diabetes, coronary artery disease, pulmonary heart disease, atrial fibrillation, hearth failure, asthma, or chronic obstructive pulmonary disease; and were prescribed with ≥3 medications at discharge. We excluded those who had a tumour, transplantation, chemotherapy, or other severe complications; were unable to understand Chinese; or were unwilling to receive medication reconciliation. Patient recruitment was done by pharmacists in sampled hospitals.

### Preliminary work

To understand the extent of medication discrepancies and common medication regimens from the sample hospitals, we first conducted a retrospective study in which our team of clinical pharmacy experts developed six categories of medication discrepancies: ‘medication duplication,’ ‘medication omission,’ ‘medication interaction,’ ‘medication addition,’ ‘inappropriate/unclear usage,’ and ‘others’ [18]. Based on these findings, we held a two-day training session for pharmacists serving the intervention group at sampled county-level hospitals, providing them with basic knowledge of medication regimen of chronic diseases, clarifying the criterion for medication discrepancy, and introducing the tailored, standardised operating procedure of conducting medication reconciliation.

### Procedures

#### Intervention groups

Patients in the intervention group received medication reconciliation by trained pharmacists in three steps (Supplement 1.3 in the **Online Supplementary Document**). Specifically, the pharmacists generated the best possible medication history for patients during patient rounds; formed a best possible medication discharge list and conducted medication reconciliation at discharge; and provided counselling for patients with a best possible medication discharge list.

During the study, 26 pharmacists performed medication reconciliation and data collection; all underwent the aforementioned two-day training. At least three pharmacists simultaneously participated in the study at each hospital.

### Control groups

Patients allocated to the control group received standard clinical treatment provided by physicians and nurses. Pharmacists were not actively involved in the medical team. Patients then received instructions regarding their discharge summary, which listed their medical diagnoses and medications they were required to take after discharge.

### Outcome

#### Primary outcome

The primary outcome was the incidence of medication discrepancy, defined as the proportion of patients experiencing at least one medication discrepancy at discharge. This outcome was evaluated by clinical pharmacy experts from our affiliated tertiary hospitals based on patients’ medical records during hospitalisation and the best possible medication discharge lists (intervention group) or discharge summaries (control group).

#### Secondary outcome

Secondary outcomes were patients’ medication adherence and health care utilisation within 30 days after discharge. These outcomes were assessed by care team members via calls on the 30th day after discharge to elicit relevant information. Patients’ medication adherence was measured using the Adherence to Refills and Medications Scale [22], with scores ranging from 12 (optimal adherence) to 48 (complete lack of adherence). Healthcare utilisation was represented by the rate of readmissions or emergency department visit within 30 days after discharge.

### Sample size

According to our previous study, we estimated that the incidence of medication discrepancy in the control arm would be approximately 60% [23]. Given a significance level of 5% and an 80% power, we needed 387 patients to detect a difference of at least 10% between the two groups. Assuming a 10% loss to follow-up and a design effect of 2, we aimed to include 1400 patients (700 in the intervention group and 700 in the control group).

### Blinding

Due to the nature of medication reconciliation, this was an open-label trail for patients and their caregivers within the trial. However, all investigators, outcome evaluators, experts from tertiary hospitals, and statisticians were blinded to the group assignment to minimise potential biases.

### Data collection and management

Pharmacists at county-level hospitals extracted clinical data from the electronic medical record systems of the sample hospitals. Data included patients’ dates of hospital admission and discharge; de-identified identification number; demographic characteristics; diagnoses; and medication information. Tertiary hospital pharmacists collected patients’ self-reported information on medication adherence and health care utilisation within 30 days after discharge via telephone survey (Table S2 in the **Online Supplementary Document**). Study investigators did not have access to the patients’ identifiable information; moreover, they were the only ones to have access to the patients’ medication utilisation data.

### Statistical analysis

In this study, we conducted intention-to-treat analyses. First, we used descriptive statistics to present patients' demographic information and incidence of medication discrepancies. We compared categorical variables (presented as counts with percentages) using χ^2^ tests. Depending on the normality of the data distribution (according to Shapiro-Wilk test), we compared continuous variables using Student’s *t*-test (normal distribution) or Mann-Whitney U test (non-normal distribution). We considered a *P*-value <0.05 as statistically significant.

We estimated the crude absolute effect of the intervention on the incidence of medication discrepancy by estimating the risk difference based on a weighted average of hospital-specific cluster-level risk differences, with weights inversely proportional to hospital-specific variances. We calculated 95% confidence intervals (CIs) for this risk difference and computed a formal hypothesis test (two-sided and at the 5% level) using a stratified *t*-test. We also fitted a logistic regression to the individual-level primary outcome data, controlling for covariates of interest, except for the treatment effect. We used the same method to analyse the readmission rate, where we presented crude and adjusted risk differences, and to analyse adherence scores, where we replaced cluster-level proportions with means in the crude analysis and computed normal linear regression instead of logistic regression to calculate covariate-adjusted difference residuals (resulting in crude or adjusted mean differences). We performed all analyses in Stata, version 16.0 (Stata Corp. LP, College Station, TX, USA).

## RESULTS

### Patients' characteristics

**Figure 1:**
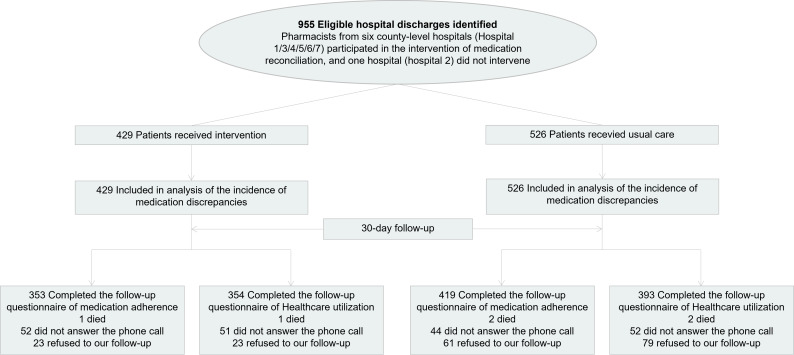
Flowchart of study participants, created per the CONSORT guidelines [19,20].

We conducted this nonrandomised controlled trial in six county-level hospitals; over the four-month recruitment period, we recruited 429 patients in the intervention group and 526 patients in the control groups (**Figure 1**). Both groups predominantly comprised male patients (intervention group: 62.0%, control group: 68.4%). The patients in the intervention group had a lower mean age than those in the control group (72.5 years, standard deviation (SD) = 7.0 vs 73.6 years, SD = 7.1; *P* = 0.012), were discharged with more medications (6.0, SD = 2.0 vs 5.3, SD = 1.9; *P* < 0.001), and had a lower average length of hospital stay (6.8, SD = 3.1 vs 7.9, SD = 4.0; *P* < 0.001). The groups did not significantly differ in the average number of complications (intervention group: 5.0, SD = 2.5, control group: 4.8, SD = 2.5; *P* = 0.244) (**Table 1**).

**Table 1:** Main characteristics of participants at discharge

Characteristics	Intervention group (n = 429)	Control group (n = 526)	*P*-value
Sex, n (%)			0.037
*Male*	266 (62.0)	360 (68.4)	
*Female*	163 (38.0)	166 (31.6)	
Age in years, x̄ (SD)	72.5 (7.0)	73.6 (7.1)	0.012
Insurance, n (%)			0.561
*UEBMI or commercial health insurance*	48 (11.2)	71 (13.5)	
*URBMI or NRCMS*	352 (82.1)	420 (80.0)	
*Out-of-pocket or medical poverty assistant*	29 (6.8)	35 (6.7)	
Hospital, n (%)			0.229
*Hospital 1*	33 (7.7)	34 (6.5)	
*Hospital 3*	88 (20.5)	83 (15.8)	
*Hospital 4*	42 (9.8)	57 (10.8)	
*Hospital 5*	102 (23.8)	116 (22.1)	
*Hospital 6*	63 (14.7)	98 (18.6)	
*Hospital 7*	101 (23.5)	138 (26.2)	
Internal medicine ward, n (%)			<0.001
*Cardiovascular*	291 (67.8)	189 (35.9)	
*Respiratory*	138 (32.2)	337 (64.1)	
Primary diagnosis, n (%)			<0.001
*COPD or hypertension*	132 (30.8)	279 (53.0)	
*CAD*	111 (25.9)	68 (12.9)	
*HF*	58 (13.5)	71 (13.5)	
*Others*	128 (29.8)	108 (20.5)	
Length of stay in days, x̄ (SD)*	6.8 (3.1)	7.9 (4.0)	<0.001
Number of comorbidities, x̄ (SD)	5.0 (2.5)	4.8 (2.5)	0.244
Number of discharge medications, x̄ (SD)	6.0 (2.0)	5.3 (1.9)	<0.001

CAD – coronary artery disease, COPD – chronic obstructive pulmonary disease, HF – heart failure, NRCMS – New Rural Cooperative Medical System, SD – standard deviation, UEBMI – Urban Employee Basic Medical Insurance, URBMI – Urban Resident Basic Medical Insurance, x̄ – mean

*Length of stay was reported by 427 patients in the intervention group and 525 patients in the control group.

### Effect of medication reconciliation

Overall, 65.7% patients in the intervention group and 79.8% of patients in the control group had at least one medication discrepancy at discharge. After controlling for potential confounders, the rate in the intervention group had an absolute risk reduction of 9.6% (95% CI = −15.6, −3.6; *P* = 0.002) in the incidence of medication discrepancy compared with the control group. We found no significant effect on the readmission rate (9.6% vs 10.4%; *P* = 0.605). We also found an improvement in adherence in the intervention group as compared to the control group, with an absolute decrease of 2.5 (95% CI = −2. 8, −2.2; *P* < 0.001) in the mean adherence score. The adjusted results showed no substantial difference with the crude results (**Table 2**).

**Table 2:** Effect of intervention on medication discrepancies, patients’ health care utilisation, and medication adherence within 30 days after discharge

Outcomes	Intervention group (n = 429)	Control group (n = 526)	Crude effect size (95% CI)*	*P-*value	Adjusted effect size (95% CI)†	*P-*value
Medication discrepancies‡						
*Incidence of medication discrepancy, n/N (%)*	282/429 (65.7)	420/526 (79.8)	−11.0% (−16.0, −6.0)	<0.001	−9.6% (−15.6, −3.6)	0.002
Healthcare utilisation§						
*Rate of readmissions, n/N (%)*	34/354 (9.6)	41/393 (10.4)	−1.0% (−5.0, 3.0)	0.590	1.3% (−3.6, 6.2)	0.605
Patients’ medication adherence¶						
*Adherence scores, x̄ (SD)*	16.6 (5.3)	19.2 (7.1)	−2.3 (−3.1, −1.5)	<0.001	−2.5(−2.8, −2.2)	<0.001

CI – confidence interval, SD – standard deviation, x̄ – mean

*Crude intervention vs control effect size estimate account for the stratified, cluster study design; we included all selected patients for the crude analyses.

†Adjusted intervention vs control effect size estimate account for the effect of patient covariates (sex, age, marriage, insurance, ward and primary diagnosis) and stratum (hospital).

‡The effect size was risk difference.

§Readmission was reported by 354 patients in the intervention group and 393 patients in the control group. The effect size was risk difference.

¶Adherence scores were reported by 353 patients in the intervention group and 419 patients in the control group. The effect size was mean difference.

### Types of medication discrepancy

In total, there were 1632 medication discrepancies identified. The average number of discrepancies at discharge per patient was 1.9 in the intervention group while that in the control group was 2.6 (*P* = 0.018). Of these, the most common discrepancy was medication omission, which occurred 394 times in 282 patients in the intervention group, compared with 814 times in 420 patients in the control group. In addition, we obtained records of medication reconciliation from four sample hospitals and found that the success rate of medication reconciliation intervention was 60.8%, and it was 100% when interfering with medication interaction, and 77.8% when interfering with medication duplication (Tables S3–4 in the **Online Supplementary Document**).

## DISCUSSION

To our knowledge, this is the first multicentre study to evaluate the effect of medication reconciliation at county-level hospitals in China. In line with prior studies, we found that pharmacist-led medication reconciliation could significantly reduce the incidence of medication discrepancies at discharge and improve patients’ medication adherence; however, this intervention did not lead to a significant reduction of health care utilisation.

Previous reviews have determined the impact of pharmacist-led medication reconciliation on medication discrepancies in several developed countries [11,24]. We also observed a significant reduction in the rate of medication discrepancies (9.6%), though a lower one than reported in studies in the US (11.7%) [25], Canada (19.9%) [26], Netherlands (29.2%) [27], Colombia (32.9%) [28], and Ireland (51.5%) [29]. One possible reason for this milder reduction was that approximately 40–80% of discrepancies documented at our sampled hospitals were clinically inconsequential [30] and thus might not have been intervened into by the pharmacists in our study. We also noted a significant improvement in adherence after discharge among patients receiving the pharmacist-led medication reconciliation intervention. At present, few studies have evaluated the effect of medication reconciliation on patient adherence across countries; those that have done so were rated as having a low certainty of the evidence [31]. A previous meta-analysis showed that pharmacist-led interventions, especially patient education, were found to have effect on improving medication adherence in older adults [32]. Thus, the improvement we observed in patient adherence may be attributable to patient counselling and education contained in the medication reconciliation intervention.

We found no significant difference in the rates of readmission or emergency department visit within 30 days after discharge between intervention and control groups, which is in line with the findings of previous studies in the UK [33], the US [34], and Sweden [35]. This might be in part due to the period of 30 days after discharge being insufficient to observe changes in the rates. After extending the follow-up period to 12 months after discharge, a randomised controlled trial in Sweden reported a 16% reduction in the rate of readmission among patients receiving pharmacist-performed medication reconciliation [36]. Another possible reason is that a one-time medication reconciliation at discharge may not be enough to impact subsequent health care utilisation. A previous study that provided comprehensive, continuous medication reconciliation since hospital admission through one full month after discharge via multiple phone calls reported a significant reduction of 13.9% in the rate of hospital readmissions within 30 days after discharge [37].

Some limitations need to be considered when interpreting our results. We planned to conduct our intervention in seven county-level hospitals, but were only able to do so in six county-level hospitals, as one did not conduct the intervention as planned. Furthermore, due to the inaccessibility of the data, we could not estimate medical costs as described in the protocol [21] and were thus unable to conduct a cost-benefit analysis. Moreover, we conducted cluster-level intervention to eliminate contamination between the intervention and control group, but there might still be unpredictable leakage between colleagues at the same hospital, possibly diminishing the difference between the control and intervention groups and thus leading to an underestimation of the true effect of our intervention. We also possibly underestimated the patients’ health care utilisation because we considered only patient readmissions and emergencies. Furthermore, although we provided a two-day training for all participating pharmacists and designed a unified implementation standard for medication reconciliation, the actual implementation of intervention measures might have varied slightly across hospitals, therefore leading to performance bias. For example, four out of six hospitals recorded in detail the subtype(s) of all medication discrepancies, while the other two only recorded whether the patients had experienced medication discrepancies. Notably, we implemented this study implemented at six hospitals, which may not be representative of all Chinese county-level hospitals. Lastly, our results might be subject to selection bias due to the nonrandomised study design and might be affected by patients’ recall bias, as patients were contacted 30 days after discharge.

## CONCLUSIONS

We found that pharmacist-led medication reconciliations at discharge reduced medication discrepancies and improved patients’ adherence at county-level hospitals in China. Future research should aim to optimise this intervention through assessing the clinical relevance of the identified discrepancies and taking a comprehensive approach towards improving clinical outcomes.

## Additional material


Online Supplementary Document
