# Supplementary material for: Evaluation of pharmacist-led medication reconciliation at county hospitals in China: A multicentre, open-label, assessor-blinded, nonrandomised controlled study
Source: J Glob Health. 2024 Apr 12;14:04058. doi: 10.7189/jogh.14.04058 (PMC11007753; doi:10.7189/jogh.14.04058)
Supplement: Online Supplementary Document [file jogh-14-04058-s001.pdf]

ONLINE SUPPLEMENTARY DOCUMENT

Title: Evaluation of pharmacist-led medication reconciliation at county hospitals in China: a multicenter, open-label, assessor-blinded, non-randomized, controlled study

Authors: Mengyuan Fu, Yuezhen Zhu, Guilin Wei, Aichen Yu, Fanghui Chen, Yuanpeng Tang, Zining Wang, Guoying Wang, Qingpeng Liu, Chunyuan Zhong, Jinghong Liu, Jie Zhong, Ping Tian, Debao Li, Xixi Li, Luwen Shi, Xiaodong Guan

1. Additional method details

1.1 Study procedure

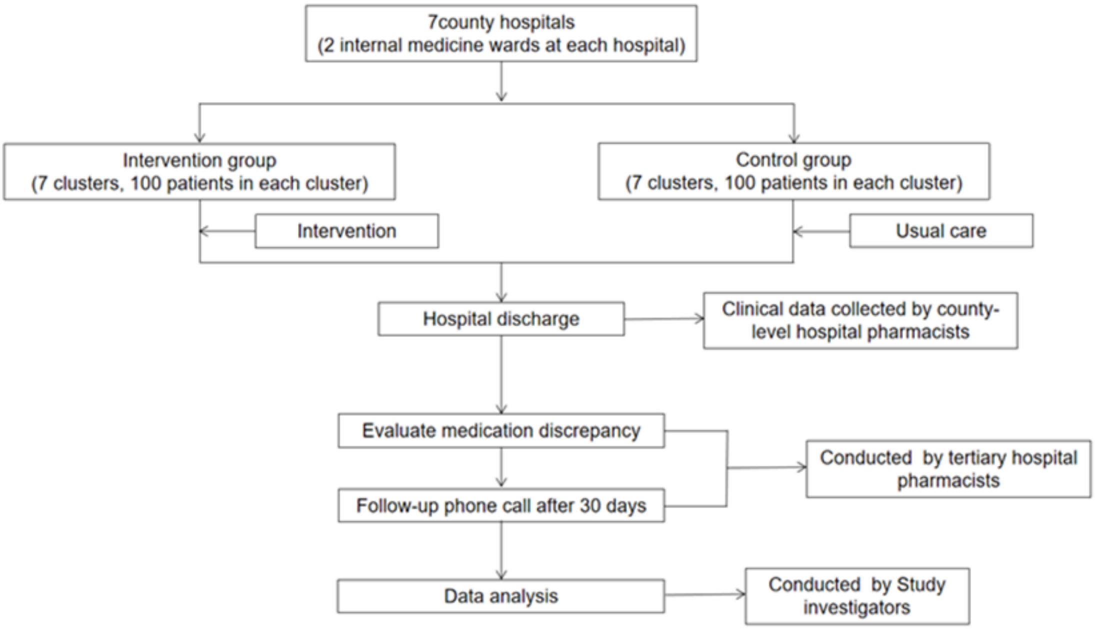

Figure S1. Flow chart summarizing the study procedure.

1.2 Preliminary work

Table S1. Training Course Schedule of pharmacists serving the intervention group.

No.	Title
1	Evaluation of the effectiveness of pharmacists-led medication reconciliation in county-level hospitals in China: implementation

	highlights of the retrospective study
2	Medical fundamentals of hypertension
3	Medical fundamentals of hypertensive emergencies
4	Pharmacological treatment of hypertension
5	Cases of pharmacological monitoring of hypertensive patients
6	Pharmacological treatment of diabetes - oral hypoglycemic agents 1
7	Pharmacological treatment of diabetes mellitus - insulin 2
8	Introduction to the treatment of antithromboembolic diseases
9	Antithrombotic therapy for coronary atherosclerotic heart disease
10	Antithrombotic therapy for atrial fibrillation
11	Bronchial asthma pharmacotherapy

1.3 Details of intervention

Patients in the intervention group received pharmacist-participated medication reconciliation. Trained pharmacists conducted medication reconciliation for patients following the three steps listed below:

Step 1: generate the best possible medication history (BPMH)

The first step of the intervention was to generate a patient's BPMH by pharmacists during patient rounds. The BPMH outlines medications that the patient actually takes before admission, including the name, dosage form, dose and admission route of each medication. This step ensured that the subsequent recommendations to simplify and optimize the medication regimen were based on full and accurate information of the patient's medication regimen. The patient's family members would be interviewed if the enrolled patient was unable to participate in the interview.

Step 2: conduct medication reconciliation at discharge

The second step of the intervention was to conduct a pharmacist-participated medication reconciliation at discharge. Pharmacists identified medication discrepancies between patient's in-hospital medication records and discharge list. Discrepancies were discussed with physicians and resolved by consensus. The pharmacists then formed a best possible medication discharge list (BPMDL). Information about medications at discharge (eg, rationale for changed medications and monitoring needs for newly initiated or stopped

medications) were summarized in the BPMDL and provided to the patient with the consent from the physician.

Step 3: provide counselling for patients

The third step was to provide patient counselling. Patients received tailored counselling conducted by pharmacists with the patients' BPMDL. The therapeutic goals and rationale for medication optimization proposal were explained and discussed in detail with each patient, as well as the benefits and potential harms of their medication treatment. Pharmacists also provided diet and lifestyle recommendations for the patients.

1.4 Follow-up table for participants

Table S2. Follow-up table for participants.

**Patient's number**	
**A Patients' medication adherence-Adherence to Refills and Medications Scale**<br>（Each item is set with responses of "none," "some," "most," or "all," of the time, which are<br>given values from 1 to 4.）	
A1 How often do you forget to take your medicine?	1. none 2. some 3. most 4. all
A2 How often do you decide not to take your medicine?	1. none 2. some 3. most 4. all
A3 How often do you forget to get prescriptions filled?	1. none 2. some 3. most 4. all
A4 How often do you run out of medicine?	1. none 2. some 3. most 4. All
A5 How often do you skip a dose of your medicine before you go to the doctor?	1. none 2. some 3. most 4. All
A6 How often do you miss taking your medicine when you feel better?	1. none 2. some 3. most 4. All
A7 How often do you miss taking your medicine when you feel sick?	1. none 2. some 3. most 4. all
A8 How often do you miss taking your medicine when you are careless?	1. none 2. some 3. most 4. all
A9 How often do you change the dose of your medicines to suit your	1. none 2. some 3. most 4. all

needs (e.g. when you take more or less pill than you're supposed to)?	
A10 How often do you forget to take your medicine when you are supposed to take it more than once a day?	1. none 2. some 3. most 4. all
A11 How often do you put off refilling your medicines because they cost too much money?	1. none 2. some 3. most 4. all
A12 How often do you plan ahead and refill your medicines before they run out?	1. none 2. some 3. most 4. all
**Adherence Scores**	
**B Healthcare utilization and medical cost**	
B1 Did you have any readmissions or emergency department visit because of the same morbidity during the past 30 days (a month)?	1.yes 2.no
B2 How much did you pay for readmissions or emergency department visits during the past 30 days (a month)?	Total expense___, Medication expense___

1.5 Data analysis (General calculations)

Percentage calculations excluded any missing prescriptions from the denominators. Summary statistics such as percentages, means and standard deviations were rounded to 1 decimal place, or 1 significant figure for numbers less than 1. Parameter estimates, including risk difference, mean difference and 95% confidence intervals (CIs), will be rounded to 1 decimal place, or 1 significant figure for numbers less than 1. All hypothesis testing were 2-sided and at the 5% significance level. All analyses were carried out using STATA Version 16.0 software. The primary and the secondary outcome analyses will be based on the intention-to-treat (ITT) population.

2. Additional trial results

Table S3. The frequency and types of medication discrepancies for patients experienced at least one medication discrepancy between intervention group

and control group.

Types	Intervention group (n=282)		Control group (n=420)		*Z*	*P*
	Total	Unit patient	Total	Unit patient		
**Total**	543	1.9	1089	2.6	-2.374	0.018
**Medication omission**	394	1.4	814	1.9		
**Medication addition**	79	0.3	175	0.4		
**Inappropriate/unclear usage**	51	0.2	55	0.1		
Inappropriate selection	19	0.1	10	0.0		
Inappropriate dosage	23	0.1	15	0.0		
Inappropriate administration time	0	0.0	10	0.0		
Inappropriate dosing frequency	6	0.0	16	0.0		
Inappropriate administration routes	3	0.0	4	0.0		
**Medication duplication**	13	0.0	42	0.1		
**Medication interaction**	2	0.0	2	0.0		
**Others**	4	0.0	1	0.0		

Table S4. Records of medication reconciliation intervention on medication discrepancies by trained pharmacists in 4 available hospitals.

Types of MDs	No. MDs	No. IMDs (%)	Success rate of MR, n (%) *
Medication duplication	14	9 (64.3)	7 (77.8)
Medication omission	63	60 (95.2)	34 (56.7)
Medication interaction	2	2 (100.0)	2 (100.0)
Medication addition	15	12 (80.0)	4 (33.3)
Inappropriate/unclear usage	34	31 (91.2)	20 (64.5)
Inappropriate selection	18	15 (83.3)	9 (60.0)
Inappropriate dosage	7	7 (100.0)	7 (100.0)
Inappropriate dosing frequency	9	9 (100.0)	4 (44.4)

Types of MDs	No. MDs	No. IMDs (%)	Success rate of MR, n (%) *
Others	15	11 (73.3)	9 (81.8)
Total	143	125 (87.4)	76 (60.8)

MDs – medication discrepancies, IMDs – intervened medication discrepancies, MR – medication reconciliation

* The intervened medication discrepancies referred to discrepancies that had received pharmacist medication reconciliation. If the final reconciliation plan proposed by pharmacists was accepted by the physician, the medication reconciliation was considered successful. Success rate of MR refers to the proportion of successful medication reconciliation times in the total intervention times (equal to the number of intervened medication discrepancies) by pharmacists.
